# Developing and Assessing the Effectiveness of a Nurse-Led Home-Based Educational Programme for Managing Breathlessness in Lung Cancer Patients. A Feasibility Study

**DOI:** 10.3389/fonc.2020.01366

**Published:** 2020-09-02

**Authors:** Aristides Choratas, Evridiki Papastavrou, Andreas Charalambous, Christiana Kouta

**Affiliations:** ^1^Nursing Department, Cyprus University of Technology, Limassol, Cyprus; ^2^Nursing Department, University of Turku, Turku, Finland

**Keywords:** breathlessness, home care, educational programme, lung cancer, nurse

## Abstract

**Introduction:** Breathlessness is the most common and refractory symptom in lung cancer patients. Even though various educational programmes have been developed, only a few were intended for implementation in the home setting for its management.

**Aim:** Feasibility of a study for implementing a nurse-led educational programme for breathlessness management of lung cancer patients at home.

**Method:** A randomized feasibility study was undertaken between February 2017 and October 2018. Patients were recruited through referral from oncologists from two oncology centers in Cyprus under certain inclusion and exclusion criteria. Patients were randomized in the intervention or control group via a computer programme, and their named family caregivers (f.c.) were allocated in the same group. Participants were not blinded to group assignment. The intervention consisted of a PowerPoint presentation and implementation of three non-pharmacological interventions. The control group received usual care. Patients were assessed for breathlessness, anxiety, and depression levels, whereas f.c. were assessed for anxiety, depression, and burden levels. F.c. also assessed patients' dyspnea level. The duration of the study process for both the intervention and control group was over a period of 4 weeks.

**Results:** Twenty-four patients and their f.c. (*n* = 24) were allocated equally in the intervention and control group. Five patients withdrew, and the final sample entered analysis was 19 patients and 19 family caregivers. In the intervention group *n* = 11 + 11, and in the control group *n* = 8 + 8. In the intervention group patients' breathlessness and anxiety levels showed improvement and their f.c.s in the anxiety and burden levels. Major consideration was the sample size and the recruitment of the patients by the referring oncologists. Attrition was minor during the study process. No harm was recorded by the participants of the study.

**Conclusions:** The study provided evidence of the feasibility of the implementation of the educational programme. For the future definitive study major consideration should be patients' recruitment method in order to achieve adequate sample size. Moreover, qualitative data should be collected in relation to the intervention and the involvement of f.c. The feasibility study was registered to the Cyprus Bioethics Committee with the registration number 2016/16. There was no funding of the study.

## Introduction

Breathlessness is a common symptom in patients with cancer (1, 2) and is the commonest amongst patients with lung cancer (3) and among patients in need of palliative care or advanced cancer (4). As classified by the American Thoracic Society in 1999 (5, p. 322), breathlessness is a subjective experience of difficulty in breathing that consists of qualitative distinctive sensations that differ in intensity. Breathlessness is caused by multiple physiological, psychological, environmental, and social factors, and simultaneously it can be exacerbated by such factors (4–6). It is a symptom that possesses great challenges for health care professionals when it comes to its effective management, especially for patients with cancer in terminal stages. With evidence showing that breathlessness increases significantly in the last 6 months of life, its frequent assessment is crucial in order for therapy to be accustomed (7). Furthermore, assessment is important for the anxiety of both the patient and their family to be identified and addressed (7).

At home, the family often undertakes the role of the caregiver for the patient, helping monitor and manage symptoms and becoming the contact person between the patient and the health care professionals (8). Family caregivers can offer limited care in relation to breathlessness management with related problems evolving, in comparison to the care offered at the hospital (9). This is mainly because family caregivers have no or limited knowledge and experience in managing breathlessness but also because of the high level of skills required to effectively manage these (9, 10). Poor management at home creates complications in patients' care affecting their quality of life and increasing admissions to the hospital. Moreover, it burdens family caregivers mainly during the end of life period of the patient, when the disease has progressed (11).

Despite the fact that the comprehensive and effective management of breathlessness remains a challenge, various strategies for managing breathlessness have been developed including pharmacological and non-pharmacological methods. For the pharmacological management of breathlessness due to cancer and the accompanying problems (e.g., anxiety, air hunger, and panic) the standard treatment is the administration of opioids with other drugs being of controversial benefit like benzodiazepines, phenothiazines, antidepressants, and steroids (12–17). There is no evidence of the effectiveness of oxygen therapy for cancer patients with dyspnea at home. Oxygen use is encouraged when saturation drops below 90% (at rest), in order to achieve improvement of functional capacity and quality of life and reduce the effects of breathlessness and mortality. However, there is the risk for patients to develop dependence (18–20).

The non-pharmacological methods include breathing techniques which assist in improving the effectiveness of the breathing cycle such as diaphragmatic breathing, inspiratory and/or expiratory muscle training, pursed-lip breathing, respiratory muscle stretching calisthenics, breathing exercises, or exercise training (stretching, walking, stairs climbing, upper, and lower aerobic) (21–25) psychoeducation, normal activities achieving training, relaxation techniques training, and psychological support (12, 15, 20, 26). The effectiveness of resistance inspiratory muscle training (IMT) was demonstrated in a two-arm, non-blinded, randomized controlled, proof-of-principle study in Cyprus and the United Kingdom in the home setting (27). The use of fans, preferably hand-held fans, directed to the face was also found to be effective (12, 15, 20, 28). Other methods include the use of mechanical ventilation techniques, e.g., CPAP, BiPAP, neuroelectrical stimulation, and chest vibration (14). Inspiratory muscle training will be used in the present study because it has already been tested for inpatient lung cancer patients in Cyprus (27). Diaphragmatic breathing technique will also be used as an already effective method (21–25) and the handheld fan, apart from its efficacy as mentioned above, because it is an economic and easy to use method for patients at home (29).

There is limited research on the effectiveness of home-based educational programmes for breathlessness and even less when is related to cancer. Olivier et al. (25) state that such programs are feasible and safe for cancer patients, so they should be assessed in association with all health care offered to cancer patients at home (if exists) in order to establish complete, holistic, and personalized home care. This was based on their study of lung or mesothelioma cancer patients undergoing chemotherapy who were offered pulmonary rehabilitation (PR) at home with exercise training, therapeutic education, and psychosocial support. The existing limited research shows that nurse-led educational programs have positive effect on patients with breathlessness due to Chronic Obstructive Pulmonary Disease (COPD), lung cancer, and heart failure (21–25, 30, 31). In the above studies the educational programs consisted of different methods of breathing retraining, pulmonary exercises, exercises for strengthening physical strength, psychosocial therapy, daily activities management training, and information in relation to the patient's disease and symptoms and their management either general for all patients or patient tailored (21–25, 30, 31).

The effectiveness of an educational intervention at home was shown in the study by Eui-Geum (30), where both the intervention and the control group received the educational programme. The intervention group received a pulmonary rehabilitation programme and the control group an educational support, and both groups showed improvement in breathlessness (30). Health related quality of life, functionality, or self-efficacy were assessed in some studies and showed improvement among the participants in the intervention group (21–23, 30, 31) except from one study (24). In the study by Olivier et al. (25), no significant improvement was shown in the breathlessness level from the intervention but did not worsen. In the study by Hermiz et al. (31) no differences among the intervention (patient tailored verbal and written education and support) and control groups (normal care) in presentation or admission to hospital or in overall functional status were noted.

The results of the above studies showed significant benefits for the intervention group in improving breathlessness not only in relation to the baseline assessment but also compared to a control group. Even though Pulmonary Function Test appeared to have no change, arterial blood gases improved and consequently breathlessness improved (23, 30). This improvement was due to the desensitization of dyspnea, the increase in vital capacity of the lungs and the decrease in the level of partial pressure of arterial carbon dioxide (PaCo_2_) (23). According to Akinci et al. (23) home-based educational programmes are preferable where no pulmonary rehabilitation programs exist at hospital level and because there is higher performance by patients as they are at home. Moreover, as Eui-Geum (30) stated, as the intensity of the program was controlled by patients, the sense of self-efficacy might improve leading to better adherence to the practical aspects of the programme. Symptom management was also improved by increasing motivation and self-care through the implementation of a nurse-led programme (21). The educational advice given on the effective breathing methods also might be the reason for improvement in breathlessness levels (22, 30). Padula et al. (30) reported that the inability to achieve secondary aims might be due to the chosen assessment tools, whereas Olivier et al. (25) stated that high attrition might have been the reason for not achieving study goals.

For the use of pharmacological and non-pharmacological methods in managing breathlessness and supporting patients and their family caregivers, nurses have the most important role either independently or as a member of a multidisciplinary team (20). Continuous nursing support is vital in the successful implementation of home care, offering the possibility to patients and family caregivers to have all the support and information when it is needed (32, 33).

In Cyprus the new cases of patients diagnosed with lung cancer are increasing every year, *n* = 198 in 2008 and *n* = 321 in 2013 (34, 35). However, no research data exist for the implementation of any programme for the management of breathless patients (due to cancer or any other disease) within or outside the hospital setting in the country.

Depending on the results of the present study, the intervention can be considered for application in a future bigger study to a broader health care area of breathless cancer patients in the home setting, for possible implementation as the first non-pharmacological intervention in the country (36). By the implementation of the programme the role of nurses in home care is expected to be enhanced and patients and family caregivers are expected to be strengthened in self-managing breathlessness at their own home setting.

The aim of the present feasibility study was the development of a nurse-led home-based educational programme for the management of breathlessness in lung cancer patients, the implementation of the programme, and the evaluation of its effectiveness to patients and their family care.

## Materials and Methods

**Figure 1:**
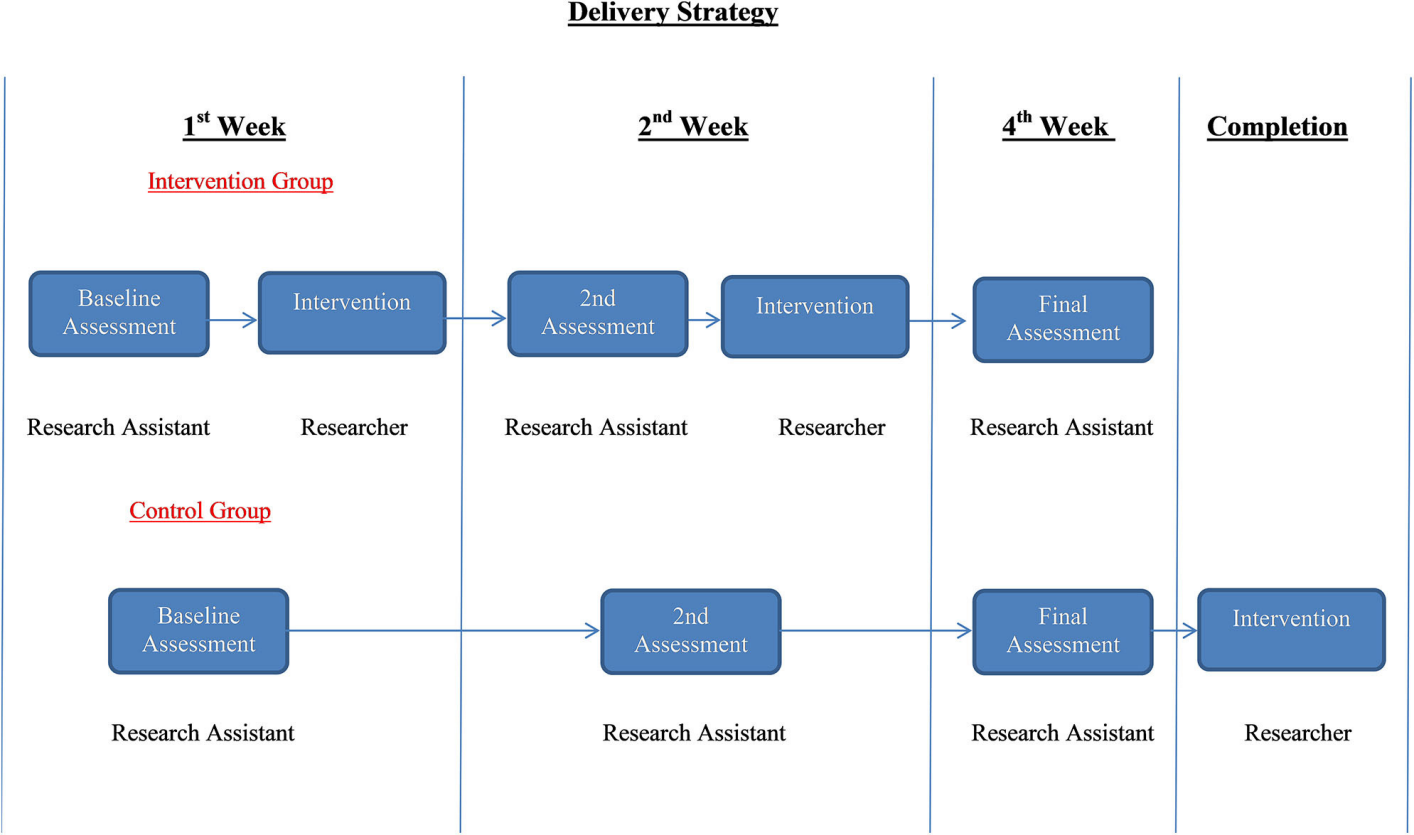
Study delivery strategy.

This was a feasibility randomized control trial that took place between February 2017 and October 2018, with an intervention group receiving the educational programme and the usual care and a control group receiving during the period of assessment only the usual care. Usual care consisted of the pharmacological management by oncologists which was prescribed to patients experiencing breathlessness, including oxygen therapy. The intervention group had a baseline assessment followed by the implementation of the educational programme. Within 2 weeks there was a reassessment and repetition of the programme, and on the 4th week the final assessment was carried out. The control group had the same assessments over the same period of time. Based on the principle of fairness, the intervention was offered to the patients that were randomized to control group following the completion of the study without any measurements recorded. Family caregivers, as named by their participating patients, were also included in the study. Together with their patients they either received the intervention or were part of the control group. Family caregivers completed their own assessment tools at the same period of time as the patients (Figure 1).

### Sample

Lung cancer patients with medium to severe breathlessness (rating 3–6 on the mBorg scale), according to an assessment from their oncologist from the two largest oncology centers in Cyprus, were eligible to participate in the study and were referred to the researcher. Other criteria for patients to be eligible for referral and inclusion in the study were (a) not to be receiving during the study period active treatment for their cancer, (b) not to be at the end of life stage in order to be able to complete the study within the 4 weeks of the study as judged by the referring oncologist, and (c) to be able to speak and understand Greek language in order to be able to understand the intervention. The last criterion was that the patient had to have a family caregiver they could name, in order to participate. Previous research studies and the number of lung cancer patients within the country were used for power analysis and calculation of the desired sample size (*n* = 45). After commencing the study doctors were unable to identify enough eligible patients to participate. Thus, the criterion for not receiving active treatment for cancer was dismissed, and patients under active chemotherapy became eligible for inclusion in the study. Moreover, it was decided to complete the study by October 2018 as a priori and regardless of the sample size.

Patients, after being informed by their oncologist about the study, if interested had to call the researcher in order to receive information about the study and give oral consent and their contact information. Participating patients had to name their family caregivers who would join them and participate in the study giving also consent, but also needed to be able to comprehend the educational material. No geographical restrictions were set. Randomization was performed allocating participants alternatively in the control or the intervention group through a computerized method by a research associate. He then contacted a specially trained research fellow, who is an experienced nurse working with patients with breathlessness, who was carrying out the assessments at patients' home setting. Participants in the study were not blinded to group assignment due to the nature of the intervention of the study. The researcher was involved in the process only for applying the intervention after being informed by the research associate carrying out measurements.

## Intervention

The intervention was designed through extensive literature review, discussions with clinical experts and the research group, and was based on the Prepared Family Caregiver model (COPE) developed by Houts et al. (37). COPE is a prescriptive problem solving model directed toward the care, information, and training family caregivers should receive in order to provide the best care at home, empowering both the patient and family caregivers (37). The educational programme included a PowerPoint presentation incorporating two video recordings and a practical exercise. The PowerPoint presentation consisted of information about the definition, causes, and clinical picture of breathlessness and its effects on patients and family caregivers. The two videos showed the effect of breathlessness on patients and the proper use of a handheld fan. The videos were used in Breathlessness Intervention Service in a hospital in UK together with an informational booklet, and their effectiveness was shown in various studies conducted for this purpose (38–40). The practical part consisted of three non-pharmacological methods for managing breathlessness and explanation of their effectiveness. Those were diaphragmatic breathing, inspirational muscle training (IMT), and use of a handheld fan. The choice for using the PowerPoint presentation was to offer complete one time information and explanation with a way that was visually interesting to the patient and which was of short duration. At the end the trainer/researcher answered questions from the participants. The educational programme lasted about 30–50 min according to patient's and family caregiver's needs. The implementation of the educational activity was undertaken by the researcher, an oncology nurse having extensive knowledge and experience in teaching patients and nurses for more than 25 years.

The educational programme was applied twice to the intervention group after the 1st and 2nd assessment and once to the control group after completing the final assessment (Figure 1).

### Data Collection

For assessing the effect of the intervention on breathlessness, data for patients were collected using the Modified Borg Scale (mBorg scale) and the Visual Analog Scale (VAS)-Breathlessness. Lung Function (FCV, FEV1) was assessed using spirometry and Hospital Anxiety and Depression Scale (HADS) was used for assessing the effect of breathlessness on patients. Data were collected for the intervention group before the intervention (baseline assessment), in 2 weeks' time and in 4 weeks' time. At all times all assessments were performed assessing the breathlessness levels, the lung function, and anxiety and depression. The same assessment tools were used to collect data from the control group at the same time intervals, in 4 weeks' time, and then the intervention was implemented to the patients in this group. Family caregivers gave data on the level they assessed their own patient's breathlessness using the mBorg scale. Also on the effect of the educational programme on them, at the same time interval as their patients, using the HADS scale for anxiety and depression and the Zarit Burden Interview (ZBI) scale for the burden they experienced. The data were collected by a specially trained research fellow.

The mBorg scale is a categorical scale which is considered the most frequently used instrument for measuring breathlessness (dyspnea) (41, 42). It requires the identification of the experienced breathlessness on a 12 point scale from 0 (no breathlessness) to 10 (very, very severe). The VAS-Breathlessness scale assesses the experienced Breathlessness at Worst and at Best and the Distress caused by the symptom. All 3 subscales rate Breathlessness or its burden from 0 (no Breathlessness) to 10 (extreme Breathlessness) over the last 24 h (43). According to Gerlach et al. (42) the VAS scales and the mBorg scale are preferred for assessing the intensity of the symptom, the quality of the sensation of breathlessness, and the related to breathing dysfunction. They support this as the scales showed concurrent validity and test/retest reliability, Cronbach's a. 0.54 (VAS) and 0.45 (mBorg) and correlations >0.8 (for both) compared to other tools (42). The choice of the above scales has taken in consideration the criteria on the choice of the appropriate tools for measuring cancer related breathlessness by Dorman et al. (44) which included among others relevance and feasibility to participants and sensitivity to changes of the symptom.

The Hospital Anxiety and Depression Scale (HADS) consists of 14 multiple choice questions assessing anxiety and depression. The questionnaire was translated into the Greek language (45) and shows internal consistency (0.87–0.85) and validity (0.722–0.749). It has been used in Cypriot cancer patients' population for assessing anxiety and depression levels (27). The same scale was used for assessing the anxiety and depression of family caregivers in a study assessing the effectiveness of a breathlessness management service (40).

The Zarit Burden Interview (ZBI) consists of 22 items, is self-completed, and assesses the burden family caregivers' experience by the caring process (46). It has been used in assessing the burden family caregivers experience during the care of patients with breathlessness due to lung cancer (47, 48). It was translated in Greek language and used in the Cypriot population showing validity and high internal reliability (Cronbach a = 0.94) (46, 49, 50). The items of the questionnaire are nine for personal strain, seven for role strain, four for relationship deprivation, and two for management of care (49). They are rated on a 5 point Likert scale from 0 (*never*) to 4 (*nearly always*). The higher the score from the sum of the items, ranging from 0 to 88, indicates greater burden (48–50).

Collection of any information on harms was not included in the study design. However, participants were informed on the consent form of the possible side effects of spirometry as well as the medical conditions restrictive of applying spirometry. Moreover, during the 4 weeks period that IMT was implemented by the intervention group participants, they were guided to report any problems faced to the researcher in order to be recorded and resolved. Patients in either group were offered support by the researcher after completion of the study for as long as they wished.

No changes in measurements took place after commencing the pilot study as the research associate did not identify any problems or difficulties during the process.

### Data Analysis

A test for equivalence of demographic characteristics of the Control and Intervention groups was performed, using chi-square test. Testing for equivalence of the Control and Intervention groups regarding the clinical characteristics of the patients (FCV, FEV1) and the level of the scales assessing Breathlessness and Anxiety/Depression and Burden for patients and family caregivers was performed using the statistic Welsch *t*-test. Measurement of the level of correlation between the clinical characteristics and the Breathlessness and Anxiety/Depression scales of the patients at baseline was performed with Pearson linear correlation. The same correlation measurement was performed for the Breathlessness, Anxiety/Depression and Burden scales of the family caregivers. The effect of the intervention on the clinical characteristics and at the level of the scales used, was performed with the statistical analysis Repeated Measures ANOVA (RM ANOVA). Specifically, the statistical significance of the Group-Time interaction coefficient for its effect on the level of clinical measurements and scales was studied. The data included in the analysis were only the data from the participants from all groups that completed the whole study.

The analysis was performed in SPSS v.21 and statistical significance was set at 0.05.

### Ethical Approval

Approval for conducting the research was granted by the Cyprus National Bioethics Committee, which is the only authorizing body for studies to be conducted in the country, with an authorization No: 2016/16. Approval was also obtained for accessing doctors and patients from the Cyprus Ministry of Health for the public hospital and the Bank of Cyprus Oncology Center. The Office of the Commissioner for Protection of Personal Data of the country gave permission for keeping records for the purpose of the study.

A written informed consent was signed by all the participants in the study (patients and family caregivers) at the first meeting with the research fellow conducting the assessments.

## Results

**Figure 2:**
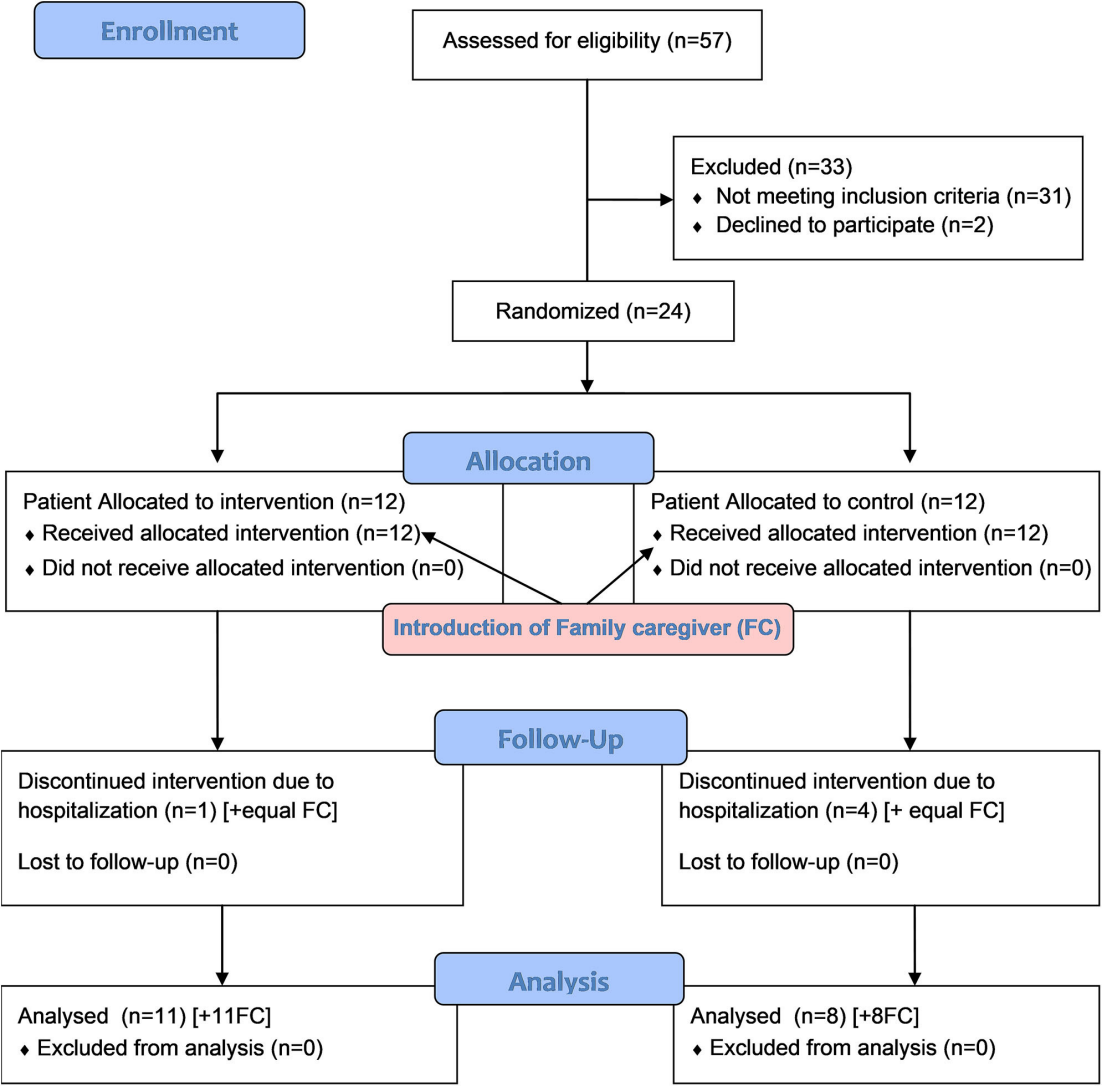
CONSORT 2010 flow diagram of the study.

Twenty-six (*n* = 26) eligible patients were referred and two refused to participate. From the 24 participating patients five (*n* = 5) withdrew from the study during the process due to hospitalization (hospice or hospital). The reason for hospitalization was deterioration of the general condition of the patients requiring inpatient care and was not related to the implementation of the intervention or participation in the study. Finally 19 patients completed the study either in the intervention group (*n* = 11) or the control group (*n* = 8). Consequently their named family caregivers were allocated in the same groups: *n* = 11 in the intervention group and n = 8 in the control group. No family caregiver expressed willingness to leave the study (Figure 2).

### Demographics

In the study the majority of the participants were male patients (*n* = 14, 74%) and between the ages of 61 and 74 (*n* = 14, 74%). Seventy-nine percent (*n* = 15) were married and 89% were Cypriots by nationality. Only *n* = 3 patients (16%) had no grammatical knowledge and the majority *n* = 11 (58%) were Gymnasium graduates (3 years in secondary school). In relation to smoking 63% (*n* = 12) of participants in both groups were smoking regardless of the gender or age group (Table 1).

**Table 1:** Demographic data of the two groups.

		**Intervention group (*****n*** **=** **11)**		**Control group (*****n*** **=** **8)**		**Total (*****n*** **=** **19)**		***p*-value**
		***N***	**%**	***N***	**%**	***N***	**%**	
Gender	Female	2	18	3	38	5	26	0.345
	Male	9	82	5	63	14	74	
Age	46–60	0	0	1	13	1	5	0.425
	61–74	9	82	5	63	14	74	
	75+	2	18	2	25	4	21	
Family	Widow	1	9	1	13	2	11	0.351
Condition	Divorced	0	0	1	13	1	5	
	Married	10	91	5	63	15	79	
	Single	0	0	1	13	1	5	
Nationality	Cypriot	9	82	8	100	17	89	0.202
	Other	2	18	0	0	2	11	
Educational	Lyceum/technical school graduate	1	9	2	25	3	16	0.504
Level	Gymnasium graduate	6	55	5	63	11	58	
	Elementary graduate	2	18	0	0	2	11	
	No elementary graduate/	2	18	1	13	3	16	
	No grammatical knowledge							
Smoking		7	64	5	63	12	63	0.96
Receiving drugs for breathlessness		3	27	1	13	4	21	0.435
Oxygen use		6	55	5	63	11	58	0.729

There was no statistically important difference in the demographic characteristics among the participants of the intervention and the control group either among the patients or the family caregivers (*p* > 0.05).

### Usual Care

Even though 21% of patients (*n* = 4) were receiving drugs for managing breathlessness, more than half (58%) (*n* = 11) were taking oxygen therapy for managing breathlessness at home. There was no statistically important difference in relation to the usual care that was received by the intervention or the control group (*p* > 0.05).

Among family caregivers 63% (*n* = 12) were females, 74% (*n* = 14) over the age of 61, and 68% (*n* = 13) were spouse/partners.

### Baseline Assessment

In the baseline assessment, for breathlessness, of both groups of patients (*n* = 19) using the mBorg scale (0–10), the median breathlessness score was 5.7 (±1.4) with a range from 3 (lowest) to 9 (highest). The median “Breathlessness at best” by the use of the VAS- Breathlessness (0–10) scale on this first assessment was 2.5 (±1.3) with 0 as the lowest and five as highest, whereas “Breathlessness at worst” of the same scale was 7.4 (±1.4) ranging from 4 to 9. Patients rated their ≪Distress due to Breathlessness≫ at 6.5 (±1) on this first assessment of the VAS-Breathlessness scale (0–10) ranging from 3 (lowest) to 10 (highest). The median Anxiety levels of the patients' baseline assessment using the HADS scale (0–21) was 8.9 (±3.7) and the median Depression levels (on the same scale) was 9.5 (±5.6) (Table 2).

**Table 2:** Baseline assessments' median of intervention and control group.

**Group**	**Both groups (*****n*** **=** **19)**		**Intervention (*****n*** **=** **11)**		**Control (*****n*** **=** **8)**		
	**Median**	**St. D**.	**Median**	**St. D**.	**Median**	**St. D**.	***p*-value**
FCV	1.9	0.5	1.96	0.54	1.78	0.3	0.373
FEV1	1.3	0.4	1.3	0.39	1.38	0.37	0.645
Breathlessness (mBorg Scale)	5.7	1.4	5.9	1.6	5.5	1.2	0.529
Breathlessness at best (VAS- Breath.)	2.5	1.3	2.8	1.6	2.1	0.8	0.239
Breathlessness at worst (VAS- Breath.)	7.4	1.4	7.6	1.2	7	1.7	0.381
Distress by breathlessness (VAS-Breath.)	6.5	1.9	7.4	1.5	5.4	1.9	0.03
Anxiety (HADS)	8.9	3.7	10.4	2.6	7	4.2	0.071
Depression (HADS)	9.5	5.6	11.3	4.5	7.1	6.3	0.472

Comparing the results of the baseline assessment between the Intervention and the Control group, there were no statistically important differences among the groups in relation to the parameters of Lung Function (FCV, FEV1), the breathlessness levels as assessed by the two scales and of the Anxiety and Depression levels (*P* > 0.05). However, in the assessment of the VAS-Distress by Breathlessness, the distress expressed by the participants was higher in the intervention group (7.4 ± 1.5) in relation to the control group (5.4 ± 1.9) (*p* = 0.03).

Linear correlations of all the variables of patients' first assessment were performed. High positive correlation was shown between patients assessment of Breathlessness (mBorg scale) and Breathlessness at Worst (VAS-Breathlessness) (*r* = 0.523, *p* < 0.01) whereas moderately positive correlation was shown between the former and Breathlessness at Best (VAS-Breathlessness) (*r* = 0.34, *p* = 0.154). Breathlessness as assessed by patients (mBorg scale) showed also high positive correlation with Distress by Breathlessness (*r* = 0.423, *p* = 0.071), moderately positive correlation with Anxiety (*r* = 0.384, *p* = 0.105), and low positive correlation with Depression (*r* = 0.279, *p* = 0.247). Moreover, Distress by Breathlessness showed high positive correlation both with Anxiety (*r* = 0.554, *p* < 0.01) and Depression (*r* = 0.503, *p* < 0.01) (Table 3).

**Table 3:** Pearsons' linear correlations of patient's baseline assessment.

	**FCV**	**FEV1**	**Breath. mBorg scale**	**Breath. at best**	**Breath. at worst**	**Distress by breath**.	**Anxiety**
FCV	1						
FEV1	,608**	1					
Breath. (mBorg scale)	−0,043	−0,26	1				
Breath. at best	−0,225	–,518*	0,34	1			
Breath. at worst	−0,24	−0,41	,523*	,559*	1		
Distress by breath.	−0,02	−0,18	0,423	,658**	,716**	1	
Anxiety	0,13	−0,277	0,384	0,342	,535*	,554*	1
Depression	0,098	−0,378	0,279	,498*	,483*	,503*	,706**

Family caregivers assessed the levels of breathlessness they believed their spouse/partner experienced with a median of 4.8 (±1.8) on the mBorg scale ranging from 2 to 9. Their median Anxiety levels at baseline assessment using the HADS scale (0–21) was 7.9 (±4.3) and the median Depression levels was 7.2 (±4.4). The burden of family caregivers was rated at 31.7 (±11.9) on the ZBI scale (0–88). Comparing the results of the intervention and the control group at baseline there is statistically important difference *p* < 0.05 in the Anxiety and Burden that family caregivers experience (Table 4).

**Table 4:** Family caregivers baseline assessments' median intervention and control group.

**Group**	**Both groups (*****n*** **=** **19)**		**Intervention (*****n***** = 11)**		**Control (*****n***** = 8)**		***p*-value**
	**Median**	**St. D**.	**Median**	**St. D**.	**Median**	**St. D**.	
Patients' breathlessness (mBorg Scale)	4.8	1.8	5.2	1.9	4.3	1.6	0.259
Anxiety	7.9	4.3	9.9	3.3	5.3	4.2	0.023
Depression	7.2	4.4	7.8	4.1	6.3	4.9	0.472
Burden	31.7	11.9	37.4	11.7	24	7	0.007

### Effect of the Intervention

Breathlessness as assessed by patients, using both scales (mBorg, VAS-Breathlessness), improved between baseline and final assessment in the intervention group whereas it deteriorated in the control group. Likewise this appeared when measuring the Distress due to Breathlessness and the Anxiety of patients where in the intervention group improved by 1.4 in both assessments and in the control group deteriorated by 1.6 and 3.3, respectively. Depression levels deteriorated during time and between the two assessments in both groups by +0.7 in the intervention group and by +1.7 in the control group. Comparing the Spirometry measurements of the two groups' Lung Function, the results in the intervention group did not show changes over the three assessments (FCV: 0.95–0.96, FEV1: 1.3–1.32) whereas in the control group the measurements appeared to show minor improvement (FCV: 1.78–1.92, FEV1: 1.38–1.5) (Table 5).

**Table 5:** Patients' measurements at baseline assessment and final assessment.

	**Intervention (*****n*** **=** **11)**		**Control (*****n*** **=** **8)**		
	**Median**	**St. Dev**.	**Median**	**St. Dev**.	***p*-value**
FCV					
Baseline assessment	1.95	0.54	1.78	0.3	0.373
4th week	1.96	0.62	1.92	0.6	0.889
FEV1					
Baseline assessment	1.3	0.39	1.38	0.37	0.645
4th week	1.32	0.43	1.5	0.69	0.523
Breathlessness (mBorg scale)					
Baseline assessment	5.9	1.6	5.5	1.2	0.529
4th week	5.1	2.6	6.4	1.8	0.228
Breathlessness at best					
(VAS-breath.)					
Baseline assessment	2.8	1.6	2.1	0.8	0.239
4th week	2.6	2.2	3.1	2.6	0.675
Breathlessness at worst					
(VAS-breath.)					
Baseline assessment	7.6	1.2	7	1.7	0.381
4th week	6.6	1.9	7.4	1.8	0.404
Distress by breathlessness					
(VAS-breath.)					
Baseline assessment	7.4	1.5	5.4	1.9	0.03
4th week	6	2.3	7	1.5	0.267
Anxiety					
Baseline assessment	10.4	2.6	7	4.2	0.071
4th week	9	4.8	10.3	3.7	0.532
Depression					
Baseline assessment	11.3	4.5	7.1	6.3	0.472
4th week	12	6.4	9.8	5.8	0.439

The statistical analysis from patients data also showed that the interaction factor Group X Time was statistically important for the Distress due to Breathlessness (F = 9.87, *p* < 0.001) and for the Anxiety (F = 5.9, *p* = 0.027) (Table 6).

**Table 6:** Time X Group interaction for the possible effect of the educational programme to the intervention group- patients.

**Measure**	**Type III sum of squares**	**df**	**Mean square**	***F***	**Sig**.
FCV	0,048	1,179	0.04	0,306	0.624
FEV	0,028	1,125	0.025	0,236	0.661
Breathlessness (mBorg Scale)	6,807	1,585	4.294	2,688	0.097
Breathlessness at best (VAS-Breath.)	3,81	1,584	2.406	1,315	0.279
Breathlessness at worst (VAS-Breath.)	4,431	1,909	2.321	1,999	0.153
Distress by breathlessness (VAS-breath.)	20,707	1,954	10.599	9,876	**<0.001**
Anxiety	49,293	1	49.293	5,9	**0.027**
Depression	8,34	1	8.34	1,181	0.292

Family caregivers' assessments showed improvement in patient's Breathlessness assessment in the intervention group (−0.6) compared to the control group (+1.5). Anxiety and Depression in the intervention group remained steady whereas it deteriorated in the control group. Burden was also deteriorated in the control group in the final assessment, but it improved in the intervention group (Table 7).

**Table 7:** Family caregivers' measurements at baseline assessment and final assessment.

	**Intervention (*****n*** **=** **11)**		**Control (*****n*** **=** **8)**		
	**Median**	**St. Dev**.	**Median**	**St. Dev**.	***p*-value**
Patients' breathlessness (mBorg Scale)					
Baseline assessment	5.2	1.9	4.3	1.6	0.259
4th week	4.6	2.7	5.8	2	0.228
Anxiety					
Baseline assessment	9.9	3.3	5.3	4.2	0.023
4th week	9.5	3.5	8.8	3.1	0.647
Depression					
Baseline assessment	7.8	4.1	6.3	4.9	0.472
4th week	7.9	4.3	8.6	4.2	0.72
Burden					
Baseline assessment	37.4	11.7	24	7	0.007
4th week	35.1	10	34.8	6.3	0.928

The interaction factor Group X Time for family caregivers' measurements was statistically important in all measurements: level of Breathlessness (*p* = 0.017), Anxiety (*p* = 0.001), Depression (*p* = 0.038), and Burden (*p* = 0.002).

The results of the study also show that there is a high positive correlation in the measurements of Breathlessness, using the mBorg scale, between the patients and the family caregivers assessments (*r* = 0.619, *p* < 0.01). This states that a high or low score in the self-assessment of breathlessness by patients relates to the same high or low score in the assessment made by their family caregiver. Moreover, it is important to note that the median level of self-assessment by the patients (5.7 + 1.14) is by one point (on the scale) higher than the assessment made by the family caregiver (4.8 + 1.8) (*t* = 1.8, *p* = 0.072). Moreover, there is high positive correlation between the anxiety patients and family caregivers experience (*r* = 0.521, *p* < 0.05) and low positive correlation in relation to depression (*r* = 0.268, *p* = 0.266).

No unintended effects or harms were recorded during the study period by any of the participants (patients or family caregivers) and regardless of the groups they were allocated to.

## Discussion

The results of this feasibility control trial and taking under consideration the small sample size revealed that the introduction of the educational program in the patients' intervention group had a moderate effect on breathlessness, on the distress due to the symptom, and to their anxiety level. Moreover, the high correlations in the first assessment between the above suggest that attempting to manage breathlessness would influence positively the distress patients experience and consequently their anxiety.

With the implementation of the educational program improvement was shown in the level of breathlessness experienced by the patients through their self-assessment. Total breathlessness level as assessed using the mBorg scale improved in the intervention group almost by one point in the 10 point scale whereas in the control group it deteriorated by the same degree. The same also happened when assessing their worst breathlessness experience. This is in line with the effect of educational interventions in managing breathlessness in previous studies either applied in the home setting (21–24) or in outpatient breathlessness clinics (38–40, 51, 52).

The implementation of the educational program did not show improvement in lung function since no significant change was identified in FCV and FEV1 measurements in the intervention group. On the contrary there was an improvement in functional capacity in the control group. These findings are in line with those reported by preceding studies by Akinci and Olgun (23) and Eui-Geum (30) regarding the results in the intervention group. However, the findings with regards to the point of improvement in the control group show it is an infrequent finding (30) and needs further study in the following larger scale study and mainly as to the possible effect of family caregiver's involvement in the process. Moreover, in the present study the influence of family caregivers in the implementation of the practical aspects of the intervention was not assessed and needs to be included in the future studies.

In addition, the distress due to breathlessness and anxiety of patients showed improvement by the intervention implemented. Respiratory distress improved in the intervention group compared to the control group which agrees with the findings of previous studies (21–24, 30, 31). Anxiety levels improvement in the intervention group is in line with the results of Olivier et al. (25) study where there was a significant improvement in stress levels. Patients' experienced depression, as assessed, does not appear to decrease during the study in both the control and the intervention groups. This is consistent with the study by Olivier et al. (25) and may be correlated with the diagnosis and treatment itself or other problems patients experience in general and not exclusively due to the process of the implementation of the educational program for managing breathlessness.

It can be argued that the confounding variables (use of oxygen and/or medication as usual care) might be the reason for the improvement of patient's breathlessness. However, as both groups received the usual care and no statistically important difference was shown between the two groups in relation to the above, the implementation of the intervention is suggestive of being the influencing component for the results of the present study.

In the present study, breathlessness appears to have a moderate to low positive association with anxiety and depression in patients, whereas discomfort due to breathlessness has a high positive correlation with both anxiety and depression. From the assessment of the respiratory distress experienced by patients, it suggests that the reported degree reflects the worst level of breathlessness experience. The above can lead to the conclusion that the consequences of the symptom and not the symptom itself are the lead causes for the patients' experienced anxiety and depression together with other factors like the diagnosis, treatment, prognosis, etc. (53, 54). Consequently if the symptom can't be managed effectively, by targeting its effects, the health care professional might be able to reduce or even prevent patient's feelings of anxiety and depression. This must be taken into consideration in the future planning of larger scale studies and explored further to lead to conclusive results, thus designing self-management home-based interventions that target both the symptom and the consequent effects.

The high positive correlation between the assessment of breathlessness by the patients and their family caregiver might have an important implication in the clinical area and the home care setting. This can be argued despite the fact that the experience is subjective and as Hui et al. (55) and Moody and McMillan (56) support, family caregivers can give accurate information to health care professionals about the status of the symptom for their patient, thus helping its management.

The low correlation of the level of breathlessness with anxiety and depression of family caregivers might imply that the degree of the symptom does not have any effect on them. On the other hand the high correlation they showed with burden might suggest that the higher the burden, the higher the anxiety and depression family caregivers experience.

The high correlation of the anxiety experienced by patients with the anxiety of family caregivers is very important for health care professionals because it implies that for every intervention applied to patients in relation to managing anxiety, family caregivers must be involved as they experience anxiety as well (57). However, the results of the present study in relation to the correlation between patients and family caregivers experience of depression, is in discrepancy with the results of the study by Li et al. (57) where there is high positive correlation in the presence of depression. This needs further study in the large research as might be due to the limitations of the present study or other factors like the culture of Cypriot cancer patients and their family caregivers.

Even though there are no data from research in the home care setting to compare the effectiveness of such an intervention on family caregivers' anxiety, depression, and burden, comparisons with studies in the palliative care setting show agreement in the improvement of the burden experienced (58, 59). However, the differences between the studies with the present in the changes shown for anxiety and depression might be due to the period characterizing palliative care (58, 59).

This feasibility study showed that the application of the educational program in the home care setting for supporting patients can be successful. Thus, should proceed with the implementation of a larger scale study taking into consideration all the problems that were faced during the present effort. The implementation of the intervention should be taken into consideration in developing health care programs for cancer patients in the community in the future. Clinical application of the educational program has potential as an adjuvant therapy, along with medication regimens for managing breathlessness for lung cancer patients and also in rehabilitation (24). Cancer nurses or home care nurses by providing the educational program might be able to achieve, through providing accurate information, support, practical advice, verbal persuasion, and gentle coaxing, the trusting environment that can be helpful to patients (24). Moreover, they need to be careful during the implementation of any supportive exercise intervention because as stated by Olivier et al. (25), in their study with cancer patients, if patients are left alone they do not adhere to the physical exercise programme due to lack of motivation. However, even by the presence of a nurse patients' anxiety can be reduced as stated by Khajian Gelogahi et al. (60) and Osterman et al. (61). In the present study, no qualitative data were collected, thus motivational issues should be taken under consideration in the future studies in order to assess whether patients were lacking motivation to practice the non-pharmacological interventions and whether family caregivers were present during the implementation of the intervention in order to help adhesion. Moreover, it needs further study in relation to the culture of the Cypriot people and family.

The measurements used for both the patients and the family caregivers appear to be suitable for the purpose of the study, and no negative issues were raised by the research associate administering them. The 4 weeks' duration for the implementation of the study, even though it did not give any negative effect, could be prolonged to 6–8 weeks. However, this could be done with caution as it might consequently lead to increased attrition due to either deterioration of patients' condition or loss of motivation by patients or family caregivers.

The positive results of the present study can be utilized for the development of a bigger scale study aiming to establish the benefit for patients with breathlessness due to lung cancer and their family caregivers, through the implementation of this educational programme. In future studies referrals by doctors and nurses of patients from other health care settings, e.g., hospice or hospital, who are going to be transferred at home, can be included in the study as well as patients in the palliative care setting.

The ultimate goal of the researchers is the implementation of the intervention as the first evidence based practice for managing breathlessness, which will be established in cancer home care nursing in the country. Moreover, the intervention might be able to expand and examine its effectiveness with breathless patients due to other chronic diseases (with the necessary modifications) like Chronic Obstructive Pulmonary Disease (COPD) and Heart Failure.

The implementation of this educational program is a step toward achieving breathlessness self-management for patients with lung cancer. This comes in line with the results of previous studies with COPD patients which promoted self-management through various interventions and resulted in improvement in breathlessness and reductions in respiratory-related hospital admissions (62).

In general the development and implementation of educational programmes for the patients and the family caregivers suggests that these can be effective, opening a new area for health care professionals (63–65).

### Study Limitations

The main limitation of the study was the small sample size preventing the generalization of the results. Despite the fact that the number of eligible patients that refused to participate was very low (*n* = 2) and the time frame of the study was prolonged to 21 months, it was very difficult to achieve a satisfactory level of participating patients.

Another major limitation of the study was the inevitable inclusion of participants that received medication for breathlessness and/or oxygen therapy. This was due to the fact that in Cyprus non-pharmacological methods are not used regularly in practice by health care professionals in order to help patients deal with breathlessness at home. In the future studies the inclusion or exclusion of patients receiving pharmacological interventions should be carefully decided by researchers. Moreover, due to the nature of the disease and of the symptom, other inclusion or exclusion criteria might be considered more carefully in order to give possibility for increasing the sample size.

## Conclusion

The results indicate that the implementation of an educational program at home for the management of breathlessness can be of benefit to cancer patients leading to the achievement of improvement in their daily living. Moreover, by establishing such educational programs, health care professionals, and mainly nurses can achieve self-management of symptoms by patients. Nurses and especially home care nurses can make the difference in addressing the problem of breathlessness encountered by patients at home and reduce unnecessary hospitalizations. They are in the ideal position to motivate patients despite the negativeness that exists due to the detrimental effects of the diagnosis and the symptom of breathlessness. It is obvious that not every patient manages to address breathlessness successfully and not every intervention is going to be beneficial for the patients; thus, careful and individualized planning is required, as stated by Akinci and Olgun (23). Moreover, it is not expected that the structured intervention will resolve the problem of breathlessness without effort by patients so there are definitely negative consequences during the process.

Nurse educators and nurse managers need to be more aware of the new era of directing nursing care into the community and into home care and should do their best to support nurses and student nurses to develop and enhance this role. This can be achieved by redesigning the systems of care implementing evidence based home care and developing nurse leaders (66).

## Data Availability Statement

The datasets generated for this study are available on request to the corresponding author.

## Ethics Statement

The studies involving human participants were reviewed and approved by Cyprus National Bioethics Commitee Independent Body by Cyprus Legislation. The patients/participants provided their written informed consent to participate in this study.

## Author Contributions

ACho: substantial contributions to the conception or design of the work, or the acquisition, analysis, or interpretation of data for the work. CK, ACha, and EP: substantial contributions to the design of the work and revising it critically for important intellectual content. All authors contributed to the article and approved the submitted version.

## Conflict of Interest

The authors declare that the research was conducted in the absence of any commercial or financial relationships that could be construed as a potential conflict of interest.
